# Neurodevelopmental outcomes of preterm babies during infancy in Eastern Uganda: a prospective cohort study

**DOI:** 10.1080/16549716.2020.1820714

**Published:** 2020-10-06

**Authors:** Gertrude Namazzi, James K. Tumwine, Helena Hildenwall, Grace Ndeezi, Paul Mubiri, Claudia Hanson, Angelina Kakooza-Mwesige, Peter Waiswa

**Affiliations:** a Maternal Newborn and Child Health Centre of Excellence, Makerere University School of Public Health, College of Health Sciences, Kampala, Uganda; b Department of Paediatrics and Child Health, School of Medicine, College of Health Sciences, Makerere University; Kampala, Uganda; c Astrid Lindgren Children’s Hospital, Karolinska University Hospital, Stockholm, Sweden; d Health Systems & Policy, Global Public Health, Karolinska Institutet, Sweden; e Department of Clinical Science, Intervention and Technology (CLINTEC), Karolinska Institutet, Sweden; f Department of Disease Control, London School of Hygiene and Tropical Medicine, London, England

**Keywords:** Neurodevelopmental disability, malnutrition, KMC, prematurity, infants

## Abstract

**Background:**

Complications due to prematurity are a threat to child survival and full developmental potential particularly in low-income settings.

**Objective:**

The aim of the study was to determine the neurodevelopmental outcomes among preterm infants and identify any modifiable factors associated with neurodevelopmental disability (NDD)

**Methods:**

We recruited 454 babies (242 preterms with birth weight <2.5 kg, and 212 term babies) in a cohort study at birth from Iganga hospital between May and July 2018. We followed up the babies at an average age of 7 months (adjusted for prematurity) and assessed 211 preterm and 187 term infants for neurodevelopmental outcomes using the Malawi Developmental Assessment tool. Mothers were interviewed on care practices for the infants. Data were analyzed using STATA version 14.

**Results:**

The study revealed a high incidence of NDD of 20.4% (43/211) among preterm infants compared to 7.5% (14/187) among the term babies, p < 0.001, of the same age. The most affected domain was fine motor (11.8%), followed by language (9.0%). At multivariate analysis, malnutrition and Kangaroo Mother Care (KMC) at home after discharge were the key factors that were significantly associated with NDD among preterm babies. The prevalence of malnutrition among preterm infants was 20% and this significantly increased the odds of developing NDD, OR = 2.92 (95% CI: 1.27–6.71). KMC practice at home reduced the odds of developing NDD, OR = 0.46, (95% CI: 0.21–1.00). Re-admission of preterm infants after discharge (a sign of severe illness) increased the odds of developing NDD but this was not statistically significant, OR = 2.33 (95% CI: 0.91–5.94).

**Conclusion:**

Our study has shown that preterm infants are at a high risk of developing NDD, especially those with malnutrition. Health system readiness should be improved to provide follow-up care with emphasis on improving nutrition and continuity of KMC at home.

## Background

Complications due to prematurity and low birth weight are the leading cause of neonatal deaths worldwide, and in 2018 they contributed about 34% of the 2.5 million newborn deaths [1]. Over 80% of preterm births and their complications are in low-income countries (LICs), in Asia and sub-Saharan Africa [2]. In Uganda, 14% of 1.6 million live births annually are estimated to be preterm [3,4]. Direct complications due to prematurity include anemia, hyperbilirubinemia with associated brain damage, feeding and breathing problems, retinopathy, and intracranial hemorrhage [5]. Those who survive may develop specific learning or behavioral impairments or reduced physical and mental health. Hence, they are unable to realize their full development potential. There is evidence showing that preterm babies have a spectrum of structural brain abnormalities that are associated with cognitive, learning and behavioral disabilities [6,7]. Survival and developmental outcomes of preterm babies are also determined by environmental factors related to birth, quality of care services and nurturing limitations as a result of maternal psychosocial effects of preterm birth [8,9]. The global Sustainable Development Goal (SDG) 3.2 target for newborn and child survival aims at reducing neonatal mortality to 12/1000 live births, or less for each country by 2030 [10]. Achieving this goal requires provision of high-quality in-patient, and follow-up care which is family centered [11]. Partnerships of skilled health providers and empowered parents who are able to participate fully in care of their preterm and sick newborn babies are critical to transforming these lives for human capital development.

Institutional deliveries have increased over time in LICs, and in Uganda over 70% of mothers deliver from health facilities [4]. Evidence-based interventions known to impact on preterm outcomes, like Kangaroo Mother Care (KMC), antenatal corticosteroids use, and newborn resuscitation are becoming more widely available. Nonetheless, access to quality neonatal care is still limited in resource-constrained settings [12–14]. In addition, there is lack of surveillance data of preterm survivors in such settings. While in high and middle-income settings there is routine clinical follow up of preterm babies [15], this is not the case in LICs. In Uganda, follow-up of preterm babies occurs at national referral hospitals and some private hospitals in the capital city. In district hospitals, where some follow up is done, neurodevelopmental assessment is not comprehensively carried out, probably due to overwhelming numbers of patients, lack of skilled health workers, and lack of validated tools and guidelines at this level of care.

The few studies conducted in sub-Saharan Africa have focused on long-term outcomes of preterm babies, usually starting in the post-infancy period [16–18]. A study in Malawi [17] among post-neonatal preterm babies revealed higher mortality, developmental disability rates, and poor growth at 18 and 24 months of corrected age. Developmental outcomes of preterm survivors in infancy are not well documented in this region. The first 6 months of life is a critical period of catch-up growth and development for preterm babies [19,20]. There is evidence of significant growth faltering at 6 months of age among infants born preterm [21]. However, there is limited information on the risk of neurodevelopmental disability (NDD) around this period. Moreover, early interventions are said to impact on physical growth, learning and social-behavioral outcomes of these infants [8,22].

The aim of the current study was to determine the neurodevelopmental outcomes of preterm infants as compared to term normal birth weight babies, and identify any modifiable factors associated with NDD among preterm infants at 6–8 months of age in eastern Uganda in order to inform interventions that can appropriately address these challenges.

## Methods

### Study design and study setting

The study utilized a prospective cohort design where preterm babies (exposed) and term/normal birth weight babies (unexposed) delivered in Iganga district hospital in Busoga region in eastern Uganda were followed up until 6 months (corrected age for the preterms) to establish their neurodevelopmental outcomes. Busoga region is the third largest region in Uganda with a population of about four million people, where over 80% reside in rural areas and depend on subsistence farming. The area is served by five general hospitals, including Iganga hospital and one regional referral hospital, with varying levels of capacity of neonatal care, with no access to neonatal intensive care or cerebral imaging.

Iganga hospital is a 100-bed capacity hospital although by the time of the study it was admitting about 200 patients. The hospital served another five adjacent districts without hospitals. The annual deliveries in Iganga hospital were 7,113 in 2018, 10% of whom were preterm and low birth weight babies admitted to the neonatal care unit (NCU). The NCU was run by three midwives, student nurses, and a pediatrician who was based on the pediatric ward and visited the unit occasionally. The unit had incubators, oxygen cylinders and concentrators, a phototherapy machine, an improvised continuous positive airway pressure (CPAP) machine and other supplies essential for neonatal care. The hospital was one of the study sites for the Preterm Birth Initiative study (2016 to 2019) which provided some of the equipment and essential supplies at the start of the intervention [23,24]. However, there was no access to surfactant to treat Respiratory Distress Syndrome, and magnesium sulphate was not in use for neonatal brain protection among women with imminent birth. In addition, there was inability to diagnose morbidities like intraventricular hemorrhage in this setting. Follow-up care of the high-risk newborn babies was carried out by midwives for 1 month, or until the child reached 2.5 kg. This did not include systematic neurodevelopmental assessment.

### Study population

The study population included babies born in Iganga hospital, who were preterm (with gestation age <37 weeks and Birth weight <2.5 kg) or term/normal birth weight babies, whose mothers were residents within a 20 km radius area of that hospital. The recruitment occurred at discharge for 3 months from May to July 2018. Neurodevelopmental assessment was carried out from November 2018 to May 2019 when the babies were 6–8 months of age (corrected for the preterm babies). We excluded all newborn babies who died before discharge and those with any congenital malformations such as spina bifida. Babies who died after recruitment were documented and reported but were excluded from the analysis for neurodevelopmental outcomes.

### Sample size estimation

A sample size of 454 participants (242 preterm-exposed and 212 term babies-non-exposed) achieving a power of 80% was computed using a sample size formula with the continuity correction (CC) factor by Fleiss [25], where we expected a risk ratio of 2.1 in neurodevelopmental disability between the exposed and non-exposed infants. Using a two-sided 95% significance level (1-alpha), with a ratio of 1:1 of those exposed to non-exposed infants, where the proportion of those with NDD among the exposed [17,26] was 23% and 11% among the non-exposed [27], a sample size of 339 (169 exposed and 169 non-exposed) was computed. Taking into consideration a 20% loss to follow up of term and preterm babies, and 10% post discharge mortality among the preterm babies [28], the sample size was raised to 454 infants, that is 212 and 242 term and preterm infants, respectively.

## Data collection

### Recruitment of the study subjects

We recruited preterm babies with gestation age (GA) <37 weeks and a birth weight of less than 2.5 kg consecutively until the sample size of 242 babies was attained. We assessed GA using the New Ballard score sheet [29] within 24 hours of birth. The New Ballard Score involves assigning of scores from −1 to 5 based on the various neuromuscular and physical maturation characteristics of the newborn baby. The total sum is then deduced to the gestation age. The total score ranges from −10 to 50 corresponding to GA of 20 to 44 weeks. For every rise in the score by 5, the GA increases by 2 weeks. For every preterm baby recruited in the study, one full-term normal birth weight baby delivered on the same day from the hospital was also recruited in the comparison cohort until the sample size of 212 was attained. We intentionally recruited more preterm babies (n = 242) than term babies (n = 212) to cater for the higher rate of mortality amongst preterm infants. All mothers who were approached agreed to participate in the study.

Three midwives (one per duty shift) employed by the hospital were trained by the first author for 2 days to identify and register the eligible preterm and term infants in the same hospital. After obtaining informed consent, a questionnaire was administered to capture mothers’ socio-demographic characteristics, the antenatal care practices including use of anti-anemia medicines (Ferrous/Folate tablets), and any maternal morbidities like malaria in pregnancy, high blood pressure, diabetes, and history of admission during pregnancy. The physical location description to aid follow-up was also captured. Records were checked for perinatal factors surrounding the birth of the baby including duration of labor, labor complications (like cord prolapse, chorioamnionitis, pre/eclampsia, etc.), and the mode of delivery. The essential newborn care within the health facility was also documented. This included birth weight, head circumference measurement, APGAR score of the baby, and KMC for the preterm babies.

### Follow-up of infants

Mothers were reached on phone at 3-months post discharge to ascertain whether the place of residence of the mother-baby pair had not changed. We employed five nurses/midwives as research assistants, supervised by the first author, for follow-up activities at 6 months of infants’ age (corrected age for preterm infants). We used the physical location description obtained from the mother either at the time of recruitment, or at 3 months (if location changed) and the telephone contacts given to locate the residence of the mother/baby pair. In some situations, babies could not be located at 6 months but at a later date due to challenges in identifying the physical location, or where mothers had travelled for various reasons, and gave appointments of home visit upon return. In other situations, the mother together with the infant had shifted from the original location to faraway places outside the catchment/study area. Such infants were considered as lost to follow-up. Similarly, those whose phone contacts were off on five attempts, and the physical locations given could not be traced, were registered as lost to follow-up.

The research assistants were trained for 5 days to conduct physical and neurodevelopmental assessment of infants at 6 months (or more) after birth (corrected age for preterms). We used the Malawi Developmental Assessment Tool (MDAT) to assess the neurodevelopmental outcomes [30]. The MDAT tool was validated in Malawi and has sensitivity and specificity of 97% and 82%, respectively, of identifying NDD among children aged 0–6 years in a rural African setting. The research assistants had previously used the MDAT tool for neurodevelopmental assessment in a community-based survey by our team [31], and an intervention study funded by Saving Brains of Grand Challenges Canada [28]. In both studies, the same research assistants were trained on how to conduct neurodevelopmental assessment by experts in that field. In the initial intervention study, they were trained by an expert midwife from Malawi who was part of the team that developed the MDAT tool. The training involved a neonatologist, the first author who was the principal investigator, and a pediatric neurologist (AKM) who continued with support supervision (including reassessment of some babies) of the Ugandan midwives. The training coupled with support supervision by the first author under the current study aimed at refreshing the midwives’ knowledge and skills in neurodevelopmental assessment of infants. A tape measure was used to assess the head circumference at birth, and this was repeated on follow up. The measurement was made by passing the tape measure over the occipital and frontal bones with all measurements recorded to the nearest 0.1 cm. In addition, we interviewed mothers on the care given to the infants since discharge from the hospital. The information captured included breastfeeding practices, KMC, any illnesses like respiratory tract infections and fevers, care-seeking practices, and immunization status. In addition, we documented information regarding home visits by health workers or community health workers, the number of clinical visits, and any readmission at the health facility.

### Data management and analysis

Neurodevelopmental assessment including Gross and Fine motor, Language and Social behavioral development were determined through use of the MDAT tool at 6–8 months’ chronological age for term infants, and corrected (adjusted) months of age for the preterm infants. Each child was scored either Pass or Fail for each domain per given age. The child was scored as fail in a given domain, if he/she failed more than two parameters of the expected at that age [30]. Data from the MDAT tool were entered using Excel while data collected using the questionnaire were entered using CSPro 7.3 software. All data were later exported to STATA version 14 and merged for analysis.

Descriptive statistics were used, stratified by term/preterm status of the infants, to determine the incidence of neurodevelopmental delay/disability. Chi-square tests were applied to categorical variables to assess any differences in maternal and infant characteristics as well as neurodevelopmental outcomes, between the preterm and term babies. In addition, odds ratios and confidence intervals were used to determine any factors associated with NDD among preterm babies.

The Z-test was used to determine the infants’ physical development in terms of weight gain for age. Those whose Z-scores were less than −2 were considered to be under weight, while between −2 and −3 were said to be having moderate malnutrition, and those whose Z-scores were <-3 were categorized as severely malnourished. The physical development data were analyzed using the two – sample t-test to assess whether there is a difference in rate of physical development in terms of the mean weight, and mean head circumference between the term and preterm babies.

### Multivariable analysis

A multivariable logistic regression was used to model the relationship between neurodevelopmental outcome among infants born preterm and the following independent factors: readmission of the infant after discharge following birth, the nutrition status (malnourished or not), KMC at home, mother’s parity, and father’s occupation as a proxy for socioeconomic status. We examined the model goodness of fit using the Hosmer Lemeshows goodness of fit test, the model was considered a good fit if the p-value was less than 0.05. Non-significant factors or predictors were dropped except for those known predictors from literature. We used the log-likelihood ratio test to compare two models that is the full model and the reduced model and the AIC/BIC criteria to select a parsimonious model. We tested for presence of multicollinearity using variance inflation factor (VIF) analyses. Multicollinearity was present if VIF was >10 or tolerance (1/VIF) was <0.1 for any independent factors in the model. A parsimonious model was presented along with model fit statistics.

## Results

**Figure 1.**
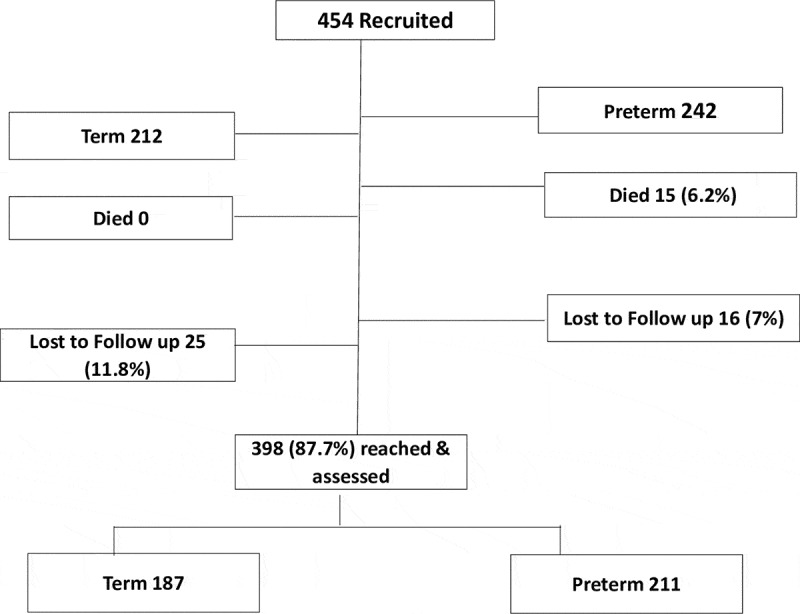
Flow diagram of infants recruited at discharge from the neonatal care unit to time of follow up and neurodevelopmental assessment at an average age of 7 months.

Of the 454 infants recruited 398 (87.7%) were reached for neurodevelopmental assessment. Fifteen (15) infants (3.3%), all preterms, died between recruitment at discharge from the health facility and at 7 months of follow up (Figure 1).

The results show that there were significant variations in maternal characteristics among mothers with preterm babies compared to those with term babies (Table 1). Specifically, the findings show statistically significant differences in prevalence of maternal perinatal and antenatal complications which were higher among mothers with preterm babies. Similarly, more (32.5%) mothers with preterm babies were admitted in hospital while pregnant as compared to 13.9% of those with term babies (p < 0.001). However, there were no significant differences regarding the mean age or level of education of the mothers.

**Table 1.** Maternal characteristics of term and preterm infants enrolled in a cohort study in eastern Uganda.

Maternal	Overall (n = 384)	Term (n = 187)	Preterm (n = 197)	p-value
Characteristics	n (%)	n (%)	n (%)	
**Age of mother, mean (SD)**	25.2 (5.4)	25.3 (5.6)	25.2 (5.2)	0.825
**Level of education of mother**				0.127
None	5 (1.3)	1 (0.5)	4 (2.0)	
Primary/7 years in school	164 (42.7)	88 (47.1)	76 (38.6)	
Secondary/11 years in School	179 (46.6)	78 (41.7)	101 (51.3)	
Advanced level/13+ years in school	36 (9.4)	20 (10.7)	16 (8.1)	
**Occupation of mother**				0.421
Salaried worker	50 (13.0)	26 (13.9)	24 (12.2)	
Business woman	109 (28.4)	53 (28.3)	56 (28.4)	
Farmer/Housewife	191 (49.7)	96 (51.3)	95 (48.2)	
Others	34 (8.8)	12 (6.4)	22 (11.2)	
**Economic status/Occupation of father**				0.423
Salaried worker	71 (18.5)	39 (20.8)	32 (16.2)	
Business man	185 (48.2)	91 (48.7)	94 (47.7)	
Labourer	34 (8.8)	12 (6.4)	22 (11.2)	
Farmer	39 (10.2)	20 (10.7)	19 (9.6)	
Others	55 (14.3)	25 (13.4)	30 (15.2)	
**Parity**				0.046**
1	135 (35.2)	72 (38.5)	63 (32.0)	
2–3	136 (35.4)	71 (38.0)	65 (33.0)	
≥4	113 (29.4)	44 (23.5)	69 (35.0)	
**Mode of delivery**				0.549
SVD	270 (70.3)	133 (71.1)	137 (69.5)	
Breech	5 (1.3)	1 (0.5)	4 (2.0)	
Caesarean section	109 (28.4)	53 (28.3)	56 (28.4)	
**Duration of labor**				0.013**
<24 hours	227 (59.1)	104 (55.6)	123 (62.4)	
≥24 hours	145 (37.8)	81 (43.3)	64 (32.5)	
Elective C/S	12 (3.1)	2 (1.1)	10 (5.1)	
**Perinatal complications**				0.008**
Yes	193 (50.3)	81 (43.3)	112 (56.8)	
No	191 (49.7)	106 (56.7)	85 (43.2)	
**Ferrous/Folic acid tablets**				0.752
Yes	374 (97.4)	183 (97.9)	191 (96.9)	
No	10 (2.6)	4 (2.1)	6 (3.1)	
**Antenatal Complications**				0.001***
Yes	313 (81.5)	134 (71.7)	179 (90.9)	
No	71 (18.5)	53 (28.3)	18 (9.1)	
**Admission during pregnancy**				0.001***
Yes	90 (13.9)	26 (13.9)	64 (32.5)	
No	294 (76.6)	161 (86.1)	133 (67.5)	

*p < 0.1, **P < 0.05, ***P < 0.001.

The study findings revealed no differences in gender between the preterm and term infants (Table 2). The mean birth weight, for the preterm babies was 1.9 kg, ranging from 0.6 to 2.4 kg, while the mean birth weight for the term babies was 3.2 kg, range 2.5 to 5.2 kg. The mean GA for preterm babies determined by Ballard score was 33 weeks, with a range of 29 to 36 weeks. There was no difference in APGAR scores among the preterm and term infants. Almost all preterm babies (91.8%) received KMC at the health facility before discharge. However, 35% of preterm babies were not breastfed within 24 hours after birth compared to 14% term babies, p < 0.001.

**Table 2.** Characteristics of preterm and term infants enrolled in a cohort study in eastern Uganda.

	Overall	Term	Preterm	
Characteristic	(n = 398) n (%)	(n = 187) n (%)	(n = 211) N (%)	P-value
**Gender**				
Male	201 (50.5)	93 (49.7)	108 (51.2)	0.772
Female	197 (49.5)	94 (50.3)	103 (48.8)	
**GA** (by Ballard score) in weeks, mean (range)	35 (29–43)	39 (37–43)	33 (29–36)	
**Type of pregnancy**				
Singleton	302 (78.6)	178 (95.2)	124 (62.9)	
Multiple	82 (21.4)	9 (4.8)	73 (37.1)	0.001
**Apgar score @5 min**				
7–10	341 (92.9)	164 (92.7)	177 (93.2)	
<7	26 (7.1)	13 (7.3)	13 (6.8)	0.851
**Immediate Breast feeding**				
Not breastfed within 24 hours	101 (25.4)	27 (14.4)	74 (35.1)	0.001
**Immediate skin to skin**				
Yes	259 (75.7)	117 (73.1)	142 (78.0)	0.292
No	83 (24.3)	43 (26.9)	40 (22.0)	
**Kangaroo Mother care at health facility**				
Yes	190 (91.8)	-	190 (91.8)	-
No	17 (8.2)	-	17 (8.2)	-
**KMC at Home**				
Yes	163 (77.3)	-	163 (77.3)	-
No	48 (22.7)	-	48 (22.7)	-
**Age of infants in months** at follow up, mean (SD)	6.9 (1.0)	7.2 (1.1)	6.6 (0.9)	0.00*
**Admissions after discharge**				
None	249 (83.3)	152 (87.4)	97 (77.6)	
Once	30 (10.0)	13 (7.5)	17 (13.6)	0.083
Twice or more	20 (6.7)	9 (5.2)	11 (8.8)	
**Any complication after discharge**				
Yes	365 (91.7)	171(91.4)	194 (91.9)	0.857
No	33 (8.3)	16 (8.6)	17 (8.1)	
**Visited health facility after discharge**				
Yes	349 (92.8)	162 (92.1)	187 (93.5)	0.586
No	27 (7.2)	14 (7.9)	13 (6.5)	
**Immunization**				
Yes	398 (100)	187 (100)	211 (100)	-
**Head circumference** (At follow up), mean (SD, Range) in cm	43 (1.7, 33.5–47)	43.8 (1.6, 33.5–47)	42.8 (1.7, 33.5–46)	0.00*

*T-test statistic.

The mean age at follow up was 7.2 months for term infants and 6.6 months corrected age for preterm babies. There were no differences in the percentage of preterm and term babies who developed complications after discharge, and those who sought care at the health facility. However, more preterm babies (22.4%) were admitted between discharge and the follow-up period compared to 12.7% term babies, p = 0.083 (Table 2).

### Growth and neurodevelopmental outcomes

The study results revealed significant differences in neurodevelopmental disabilities between term and preterm infants, with higher incidence of NDD among preterm babies (at 6.6 months of corrected age): 20.4% compared to 7.5% among term babies (p < 0.001). The disabilities among preterm infants were mostly in the Fine motor domain (11.8%), followed by Language development (9.0%), while among the term infants the disabilities were predominantly in the Social development domain (4.8%). There were no significant statistical differences in disabilities in the Social domain across the two groups of infants (Table 3).

**Table 3.** Physical and neurodevelopmental outcomes of preterm and term infants in eastern Uganda.

	Overall (N = 398)	Term (n = 187)	Preterm (n = 211)	
	n (%)	n (%)	n (%)	P-value
**Developmental outcome**				
Failed Gross motor	15 (3.8)	1 (0.5)	14 (6.6)	0.001
Failed Fine motor	31 (7.8)	6 (3.2)	25 (11.8)	0.001
Failed Language	21 (5.3)	2 (1.1)	19 (9.0)	0.001
Failed Social behavior	23 (5.8)	9 (4.8)	14 (6.6)	0.437
Neurodevelopmental Delay/Disability (NDD) (Failed at least one domain)*	57 (14.3)	14 (7.5)	43 (20.4)	0.001
Failed 3+ domains	8 (2.0)	1 (0.5)	7 (3.3)	0.048
**Physical Growth outcome**				
Prevalence of underweight (WAZ<-2)	43 (10.8)	4 (2.1)	39 (18.6)	0.001
Moderate (waz≥-3 & waz<-2)	31 (7.8)	4 (2.1)	27 (12.9)	0.001
Severe malnutrition (waz<-3)	12 (3.0)	0 (0.0)	12 (5.7)	0.001

*Some infants had neurodevelopmental disability in more than one domain.

Similarly, there were significantly more infants with malnutrition as demonstrated by Z-score of less than −2 of weight for age among the preterm infants (18.6%) as compared to the term infants (2.1%), p = 0.001. Severe malnutrition was seen in 5.7% among preterm infants while there were no cases of severe malnutrition identified among the term babies.

### Modifiable factors among preterm infants

At bivariate analysis, malnutrition was found to be significantly associated with NDD, with 32.5% of cases with malnutrition having NDD (Table 4), OR = 2.26 (95% CI: 1.05–4.88). Malnutrition was significantly higher among male infants (p = 0.008), very low birth weight babies (p = 0.000), those who were not exclusively breast fed (p = 0.001) and whose fathers were laborers as compared to salaried workers (0.045). However, the socioeconomic status, birth weight, and breastfeeding practices among preterm babies were not independently associated with NDD. There was weak evidence that KMC at home after discharge was associated with less odds of developing NDD (OR = 0.53; 95% CI: 0.25–1.10). The mean number of times the babies were put in KMC position at home were 2.8, with each session lasting 1.6 hours on average. KMC at health facility before discharge was not associated with NDD. Similarly, maternal characteristics such as education status, parity and maternal morbidities were not associated with NDD among preterm infants (Table 4)

**Table 4.** Bivariate analysis of modifiable factors associated with NDD among preterm infants in eastern Uganda.

	No NDD	NDD		P-value
Modifiable factors	n (%)	n (%)	COR (CI)	
**Birth weight in kg**				
<1.5	23 (71.9)	9 (28.1)		
1.5 to 1.99	54 (79.4)	14 (20.6)	1.13 (0.32–4.03)	0.846
2.0 to 2.5	91 (82.0)	20 (18.0)	0.71 (0.17–2.85)	0.626
**Malnutrition Z-Score <-2**				
No	141 (82.5)	14 (6.6)		
Yes	27 (67.5)	13 (11.8)	2.26 (1.05–4.88)	0.035**
**KMC at home**				
No	34 (70.8)	14 (29.2)		
Yes	134 (82.2)	29 (17.8)	0.53 (0.25–1.10)	0.086*
**KMC at health facility**				
Yes	150 (78.9)	40 (21.1)		
No	15 (88.2)	02 (11.8)	1.99 (0.44–9.11)	0.370
**Exclusive breast feeding**				
Yes	107 (81.1)	25 (18.9)		
Mixed Feeding	61(77.2)	18 (22.8)	1.2 (0.63–2.49)	0.503
**Readmission of infant after discharge**				
No	69 (71.1)	28 (28.9)		0.083*
Yes	19 (67.9)	9 (32.1)		
**Mothers’ education**				
O-level (11 years of school) or less	86 (78.9)	23 (21.1)		
A-level (12 years)+	12 (70.6)	5 (29.4)	0.58 (0.19–1.75)	0.34
**Economic status: Father’s occupation**				
Salaried	26 (72.2)	10 (27.8)		
Business	79 (79.0)	21 (21.0)		
Farmer/unemployed	63 (84.0)	12 (16.0)		0.346
**Parity**				
1	54 (81.8)	12 (18.2)		
2	59 (81.9)	13 (18.1)	0.99 (0.42–2.36)	0.985
3+	55 (75.3)	18 (24.7)	1.47 (0.64–3.34)	0.356
**Complications during pregnancy**				
Yes	50 (75.0)	5 (25.0)		
No	153 (80.1)	38 (19.9)		0.590
**Admission in pregnancy**				
Yes	50 (74.6)	17 (25.4)		
No	118 (81.9)	26 (18.1)		0.219
**Maternal perinatal complications**				
Yes	74 (77.9)	21 (22.1)		
NO	94 (81.0)	43 (19.0)		0.573

*p < 0.1, **P < 0.05, COR = Crude Odds Ratio, NDD = Neurodevelopmental disability.

The results at multivariate analysis revealed a good fit for the model of the relationship of five independent factors and NDD among preterm infants, Hosmer-Lemeshow’s goodness of fit test, LR-chi of 15.71, p = 0.028 (Table 5). Malnutrition was significantly associated with increased odds of developing NDD, AOR = 2.92 (95% CI: 1.27–6.71). On the other hand, KMC at home was associated with reduced odds of developing NDD, AOR = 0.46 (95% CI: 0.21–1.00). Readmission after discharge of infants born preterm, a sign of severe illness, was associated with increased odds of having NDD but this was not statistically significant.

**Table 5.** Multivariate logistic regression of key factors associated with neurodevelopmental disability among preterm infants in eastern Uganda.

Covariate		AOR (95% CI)	p-value
Re-admission of baby after discharge	Yes	2.33 (0.91–5.94)	0.077
	No	1.0	
Malnutrition, Z-Score <-2	Malnourished	2.92 (1.27–6.71)	0.012
	Normal weight for age	1.0	
KMC at home	Yes	0.46 (0.21–1.00)	0.051
	No	1.0	
Parity	1–2	0.62 (0.26–1.44)	0.268
	3+	1.0	
Father’s occupation	Business	1.0	
	Salaried	2.20 (0.86–5.67)	0.101
	Laborer/farmer	0.63 (0.27–1.43)	0.268

N = 211, LR-chi2 = 15.71, P-value = 0.028, AOR = adjusted odds ratio.

## Discussion

This is one of the first studies in this setting to determine the neurodevelopmental outcomes and modifiable factors among preterm infants. The findings revealed a high incidence of NDD of 20.4%, and severe disability of 3.3% associated with malnutrition among preterm infants at 6.6 months of corrected age, as compared to 7.5%, and 0.5%, respectively (p = 0.001), among term normal birth weight babies. The most affected developmental domain among preterm infants was Fine motor, followed by the Language domain. There was no statistically significant difference in social behavioral development among preterms and term babies. This may be explained by the similarities in parental engagement and stimulation of these infants irrespective of GA at birth. These findings are important as they suggest that mortality, NDD and malnutrition are higher and occur early in preterm infants after hospital discharge in our setting.

Our findings are interestingly similar in the domains affected to those of Do et al. (2019) in Vietnam [32] where preterm babies at 2 years had poor cognitive, language and motor development as compared to term healthy babies. This could indicate that without effective corrective interventions, NDD among preterm babies does not change much after the infancy period. However, in that study social development of those infants was not reported, probably due to the limitations of the assessment tool that was used. In our study, developmental disability among term infants was mainly in the social behavioral domain which is consistent with the findings of our population-based study, confirming that parent–child interactions for optimal development are a challenge in this setting [31].

Although Gladstone et al. (2011) did not find any significant neurodevelopmental differences among term and preterm babies at 12 months, significant differences were reported at 18 months of corrected age [17]. Others have reported significant NDD among preterm babies during infancy irrespective of the GA [9,18,33]. Preterm babies may experience intraventricular hemorrhage, hypoxic-ischemic encephalopathy and/or seizures which raise the risk of developmental disabilities including motor and speech among these children [7]. Thus, quality of care in neonatal care units is critical in reducing the rate of NDD for preterm babies. However, neonatal intensive care units in most LICs are not yet widely available and where they exist the quality of care is still a challenge due to various health system bottlenecks [14].

We found that malnutrition was the main factor associated with NDD among preterm babies. About 20% of preterm infants had malnutrition, of whom a third had NDD. Malnutrition due to poor feeding, infections, and/or poor gut development increases the vulnerability of preterm babies to developing NDD [34,35]. Childhood malnutrition in LICs is still a major challenge to early childhood development [36]. Poor nutrition and growth have been associated with NDD [37–40]. Early interventions such as increased nutrition and breast milk fortification do not only reduce mortality but are also critical in influencing the trajectory of development among these infants. Early identification of those at greatest risk of poor health outcomes in order to allow appropriate and targeted follow-up among preterm infants is of utmost importance for better health outcomes, even later in life [22]. In this study, about 20% of preterms did not breastfeed within 24 hours after birth and exclusive breastfeeding was not done for 35% of the preterm infants in the first 6 months. Although breastfeeding practices were not directly associated with NDD among preterm infants, they were significant predictors for malnutrition. In light of the high incidence of malnutrition, concerted efforts are needed in ensuring proper and adequate feeding of preterm babies with special focus on those who are unable to suckle in this setting. Introduction of breast milk banks, and fortification of breast milk to meet the growth needs of very preterm babies should be considered. Nurses/Midwives also require skills and competencies in feeding the sick and small/preterm babies. In addition, parents and guardians can be educated on proper feeding for these infants and the importance of KMC at home, while their babies are still admitted in the health facility.

Although almost all preterm infants had KMC at the health facility, only 77% continued KMC practice at home for 4.5 hours a day on average. KMC is effective in reducing morbidity, and improved growth and neurodevelopment [41–44]. A Cochrane review by Conde-Agudelo et al. in 2011 found that infants who received KMC had reduced infection rates, increased breastfeeding, better infant growth as demonstrated by increased head circumference and improved mother-infant attachment [43]. On the other hand, community KMC did not have an effect on infants’ neurodevelopmental outcomes in India [45], probably due to late initiation following birth.

Policy and program implications of this work include: as we improve preterm survival, we should be aware of NDD with its social, health system, and economic implications. Systematic follow-up of these infants for nutrition, growth and neurodevelopment monitoring until at least catch-up growth is recommended [46], to accelerate reduction in not only child mortality but also development and transformation of human capital. However, this comes with financial and logistical implications for the provision of quality and comprehensive health-care services for these infants, which should be considered for the health system in Uganda and other LIC settings.

### Methodological considerations

The study involved only one district-level hospital, hence limiting generalizability of the findings to the preterm babies in the whole country. However, this particular hospital is a typical district hospital in the country though may be considered to have had a higher-level quality of care compared to others in the country at that time as it had concerted capacity building efforts for newborn care under the Preterm Birth Initiative, meaning that findings elsewhere might be worse. In addition, we used the Ballard score for GA estimation due to lack of first trimester/early ultrasound scan (USS) during pregnancy for most of the mothers which may limit the accuracy of gestation age dating. In this setting, there were no mechanisms of diagnosing intraventricular hemorrhage, one of the commonest causes of disabilities among preterm babies, and no preventive treatment modalities for this complication. Despite these limitations, the findings are still useful since malnutrition can be prevented in this setting. Although the MDAT tool used has some areas of personal care, it is currently the only developmental assessment tool we know of which was developed and validated for rural African setting. In this study, we had an attrition rate of 12.3%. In estimating the sample size for our study, we took care of loss to follow-up by adjusting the sample size by more than 20% for the preterm babies. Therefore, the attrition rate of 12.3% would have very minimal or no effect on our results.

## Conclusion

Preterm infants in the study setting are at a high risk of developing neurodevelopmental disabilities. Under-nourished infants and those whose mothers did not consistently practice KMC at home were more likely to have NDD. Health system readiness should, therefore, be improved to provide systematic follow-up care, inclusive of growth and neurodevelopmental assessments, to prevent and address malnutrition. Increased nutrition support, and continuity with KMC at home after discharge from health facility can be helpful in curbing NDD among preterm infants in low resource settings.

## Paper context

Available literature show that preterm infants are at risk of developing neurodevelopmental disabilities. However, the incidence and associated factors in low-income countries are not clear. This study revealed a high neurodevelopmental disability of 20.4% associated with malnutrition among preterm infants. Kangaroo Mother Care at home was found to reduce the risk of neurodevelopmental disability. Therefore, health system readiness should be improved for follow-up care, improved nutrition and Kangaroo Mother Care for preterm infants.
